# Examining Oral Hygiene Behaviors, Oral Health-Related Quality of Life, and Attitudes Toward Oral Health Among Minority Students

**DOI:** 10.7759/cureus.60209

**Published:** 2024-05-13

**Authors:** Deonschae Lyons, Angelina Zarzeczny, Payal Kahar

**Affiliations:** 1 Department of Biology, Florida Gulf Coast University, Fort Myers, USA; 2 Department of Health Sciences, Florida Gulf Coast University, Fort Myers, USA

**Keywords:** oral health attitudes, ohrqol, self-reported oral health, college students, minority

## Abstract

Purpose: This study aimed to examine the association between oral hygiene behaviors, oral health-related quality of life (OHRQoL), oral health attitudes, and self-reported oral health problems among minority undergraduate students attending a state university in Florida.

Methods: Chi-square analysis was conducted to examine the differences in self-reported dental caries and bleeding gums by oral hygiene behaviors, daily habits, and past oral experiences. Mann-Whitney U test was conducted to compare OHRQoL and attitude items with self-reported oral health issues and demographics.

Results: A greater percentage of students (54.5%) who brushed for ≤1 minute experienced gum bleeding compared to 45.5% who did not report gum bleeding (*p *= 0.005). Median values for difficulty biting or chewing foods, took days off school, difficulty doing usual activities, and pain were significantly higher among those with self-reported dental caries and bleeding gums. Difficulty with speech significantly varied with the presence of bleeding gums and teeth insecurities (*p =* 0.027 and *p =* 0.011, respectively). Avoiding smiling experienced pain was significantly different among with teeth insecurities (*p =* 0.001, *p =* 0.031). Of the various attitude statements, “I value keeping my mouth healthy” significantly varied with dental caries and bleeding gums (*p =* 0.002; *p =* 0.005). Attitude toward acceptance of age-related tooth loss was significantly different with age (*p = *0.022).

Conclusions: The results provide evidence of self-reported oral health problems affecting OHRQoL and attitudes toward oral health. Improving oral hygiene behaviors with resources available for regular dental visits to minimize dental issues and improve OHRQoL among minority students is warranted.

## Introduction

Many college students are likely aware of the influence of lifestyle choices on health, yet the oral health of minority students is often overshadowed due to barriers to accessible dental health information and services. In general, 91% of adults between the ages of 20-64 years had dental caries and 27% had untreated tooth decay in 2011-2012 [1]. Although tooth decay is less prominent in the general population, people of color are twice as likely (42%) to suffer from untreated tooth decay than non-Hispanic whites (22%) due to low rates of dental visits [2]. Minority populations are more likely to experience a decreased oral health-related quality of life (OHRQoL) as tooth decay caused by poor oral hygiene may impact sleep, eating, and social interactions [3]. Poor oral health has also been linked to lower academic performance and missed school days due to decreased quality of sleep from tooth pain [4-5]. OHRQoL is closely aligned with quality dental care, and barriers to preventive services may severely impact a student’s wellbeing, both physically and emotionally [6]. Furthermore, OHRQoL goes beyond pain or discomfort while eating as it can lead to unprecedented effects on an individual’s self-image and relationships with others [6].

Due to this, it is important for healthy oral patterns to be established in early childhood to help enforce these preventative behaviors into adulthood. This is particularly relevant to college students as research has discovered that those entering young adulthood participate in unhealthier eating habits, such as irregular meals, “junk food” consumption, and a reduced intake of nutritious foods like fruits and vegetables due its convenience or affordability [7]. In addition, preexisting oral issues may influence oral perceptions and habits. Adolescents with poor oral health, for example, displayed negative attitudes and unhealthier oral behaviors yet perceived their oral health to be “good” [8].

Overall, healthy oral practices should be integrated into students’ daily routines and executed effectively to reduce the risk of unfavorable health issues. For example, oral health issues may contribute to food avoidance and poor dietary quality as individuals may resort to softer, chewable foods over those that may trigger tooth pain [9]. Not only that, but individuals with perceived halitosis, or bad breath, have noted avoiding social interaction and losing friends due to poor oral health [10-11]. Therefore, oral preventative measures such as brushing twice a day, avoiding sugary food and drink consumption, flossing after every meal, and regularly visiting the dentist are crucial to maintaining healthy teeth and reducing the risk of severe dental complications, but this information may not be readily accessible to all populations or may not be prioritized due to inconvenience and expense [12]. In fact, a study conducted in Northern Manhattan found that dental hygiene was poorer in minority communities than in other American communities across the United States [13]. The study also demonstrated a high rate of dental caries and plaque buildup in minority adolescents, which implies that oral hygiene habits are not being readily enforced starting in childhood. Yet, while oral health is a challenge faced throughout the United States, some areas experience greater barriers to accessibility to quality preventive care. In Florida, for example, access to dental care is limited by a shortage of dental professionals and poor Medicaid acceptance, which greatly affects marginalized populations since few dentists opt to work in underserved and rural communities due to poorer benefits [14]. Likewise, given the state’s poor accessibility of dental care, minority college students in Florida may feel discouraged from pursuing dental services or advocate for their own oral health if they cannot afford it or are unaware of the consequences untreated dental issues may pose [15].

Given the prevalence of poor dental hygiene and dental issues in minority populations and Florida’s reputation for poor oral health, this study was conducted to examine oral hygiene behaviors, OHRQoL, dental care, attitudes, and self-reported oral health problems among minority college students. Moreover, at the university where the study was conducted, the minority population makes up 37% of the student population [16]. Increasing research on this topic would not only serve to further understanding of how to eliminate oral health disparities but also highlight the importance of recognizing oral health as an integral component of one’s general well-being.

## Materials and methods

Research design and procedure

This cross-sectional study was conducted at Florida Gulf Coast University in Fort Myers, Florida, in the spring of 2022. Following approval from the institutional review board committee (Florida Gulf Coast University Office of Research and Sponsored Programs, per 45 C.F.R. 46.110), anonymous surveys were conducted; recruitment for the study was attempted through email, distributing flyers with a QR code throughout campus, and by person-to-person solicitation. Before taking the survey, each participant was informed of the details of the survey and asked to sign the consent form.

Participants

Participation in the survey was completely voluntary. Inclusion criteria included minority students who self-identified as undergraduate minority students at Florida Gulf Coast University. These were students who self-identified themselves as Black (or African American), Hispanic (or Latino), Asian, Native American, Pacific Islander, or two or more races and were 18 years of age or older. Approximately 450 students were approached, of which 151 participated in this study.

Measures

Demographics

Information was collected on age, gender, and race/ethnicity. Age was treated as a categorical variable and subdivided into two groups: 18-24 years and 25 years or older. While the age of college students typically ranges between 18 and 24 years, maturity of brain and craniofacial structures and re-emergence of malocclusion (if corrected during adolescence) take place between 20 and 25 years. Moreover, age groups 20-34 years have the highest untreated dental caries [17]. Minority students were grouped as Asian or Asian American, Hispanic/Latino, Black, and bi/multiracial regarding their race/ethnicity.

Oral Health-Related Measures

The surveys collected information on oral hygiene behaviors, such as brushing frequency, length, use of floss and other dental hygiene tools, consumption of sugary foods, and visits to the dentist. Data were also collected on past oral health experiences such as supervision by an adult while brushing, allowance of sugary foods, and experience with dental caries as an adolescent. Self-reported oral health status, untreated dental caries, bleeding gums on brushing and flossing, and teeth insecurities (crooked teeth, gaps between your teeth, stained teeth, chipped/broken teeth, or smell) were also obtained from the respondents. OHRQoL was determined using seven questions on a four-point Likert scale (never, rarely, occasionally, and very often). Questions included how often you have experienced difficulty with chewing, trouble with speech, avoided smiling, dry mouth, and pain, took days off school/work, and had difficulty doing usual activities due to your mouth and teeth in the last 12 months were derived from the Oral Impact on Daily Performance Index [18]. These items indicated high internal consistency (Cronbach’s alpha: 0.80). Attitudes toward oral health, dental care and changes to oral health with aging were also elicited using five questions on a five-point Likert scale (strongly disagree to strongly agree). These questions were developed by the American Dental Association’s Health Policy Institute [19].

Data Analysis

Complete data were entered into an SPSS database (IBM Corporation, version 28, Armonk, NY). Descriptive statistics such as age, gender, and race/ethnicity are presented as numbers and percentages. Chi-square analysis was conducted to examine the differences in self-reported dental caries and bleeding gums by oral hygiene behaviors, daily habits, and past oral experiences. Finally, a Mann-Whitney U test was conducted to compare OHRQoL and attitude items with self-reported oral diseases and demographic characteristics, such as gender and age. Median and the interquartile range (IQR) for each item with respect to self-reported oral health problems are also reported.

## Results

Table 1 represents the demographics of the minority students who participated in the survey. Nearly 80% of the participants were within the age group of 18-24 years. Females accounted for 73.5% of the sample. Race/ethnicity was subcategorized into Black/African American (41.7%), Hispanic/Latino (37.1%), Asian (7.9%), and biracial (9.3%). In addition, the distribution of oral health and dental care attitudes shows that most students valued oral health (91.4%), felt they needed to visit the dentist twice a year (77.1%), and agreed that regular visits will help keep them healthy (83%).

**Table 1:** Demographics and attitudes toward oral health and dental care

Variable	N	Percent
Age (years)		
18-24	120	79.5
25 and older	26	17.2
Gender		
Male	36	23.8
Female	111	73.5
Race/Ethnicity		
Asian or Asian American	12	7.9
Hispanic/Latino	56	37.1
Black	63	41.7
Bi/multiracial	14	9.3
I value keeping my mouth healthy.		
Strongly agree-agree	128	91.4
Neither agree nor disagree	1	0.7
Strongly disagree-disagree	11	7.9
Regular visits to the dentist will help keep me healthy.		
Strongly agree-agree	112	83
Neither agree nor disagree	15	11.1
Strongly disagree-disagree	8	5.9
As I grow old, I accept that I will lose some of my teeth.		
Strongly agree-agree	66	49.6
Neither agree nor disagree	21	15.8
Strongly disagree-disagree	46	34.5
I need to see the dentist twice a year.		
Strongly agree-agree	101	77.1
Neither agree nor disagree	18	13.7
Strongly disagree-disagree	12	9.1
It is easier to get ahead in life if I have straight bright teeth.		
Strongly agree-agree	72	54.9
Neither agree nor disagree	32	24.4
Strongly disagree-disagree	27	20.6

Table 2 presents oral hygiene behaviors and past oral health experiences by self-related oral health problems. Dental caries were categorized into two groups: students reporting no dental caries and students reporting the presence of dental caries. Similarly, bleeding gums were categorized into two groups: those who experienced gum bleeding while brushing and flossing and those who did not. A greater percentage of students (54.5%) experienced gum bleeding compared to 45.5% who did not report gum bleeding when they had brushed for one minute or less, and this difference was statistically significant (χ2 = 7.9; *p *= 0.005).

**Table 2:** Oral hygiene behaviors and past experiences by self-reported oral health problems * *p*-value significant at alpha 0.05

Self-reported oral health issues	Dental caries				Bleeding on brushing/flossing			
	Yes (1-3)		No		Yes		No	
	N	%	N	%	N	%	N	%
Brushing frequency								
Once	12	50	12	50	12	50	12	50
Twice or more	52	41.9	72	58.1	47	37.9	77	62.1
Length of brushing (minutes)								
One or less than one	22	40	33	60	30	54.5	25	45.5
Two or more	42	45.2	51	54.8	29	31.2	64	68.8*
Yearly visits to the dentist								
Once	21	36.2	37	63.8	28	48.3	30	51.7
Twice or more	33	45.8	39	54.2	24	33.3	48	66.7
No visits	9	50	9	50	7	38.9	11	61.1
Last dental visit								
Less than a year	36	42.4	49	57.6	30	35.3	55	64.7
1-2 years	20	45.5	24	54.5	20	45.5	24	54.5
≥3 years	8	40	12	60	9	45	11	55
Supervision while brushing when young								
Yes	44	38.9	69	61.1	41	36.3	72	63.7
No	16	59.3	11	40.7	15	55.6	12	44.4
How often are parents allowed to consume sweets								
Very often	14	38.9	22	61.1	15	41.7	21	58.3
Occasionally-rarely	45	32.4	58	56.3	40	38.8	63	61.2
Dental caries as adolescents								
Yes	40	47.1	45	52.9	37	43.5	48	56.5
No	24	38.7	38	61.3	21	33.9	41	66.1

Table 3 presents the difference between self-reported oral conditions and problems due to the conditions of the mouth and teeth. Median values for difficulty biting or chewing foods were higher among those with dental caries and bleeding gums, and these findings were statistically significant (*p *= 0.002 and *p *= 0.005, respectively). Median values for difficulty with speech were significantly higher with the presence of bleeding gums and teeth insecurities (*p *= 0.027 and *p *= 0.011, respectively). Median values for dry mouth among those who reported dental caries were higher than those who did not, and these findings were statistically significant (*p *= 0.03). Avoiding smiling was significantly different among those with teeth insecurities with a *p*-value of 0.001. "Took days off school" was significantly different with the presence of dental caries (*p* = 0.004) and bleeding gums (*p *= 0.045). Difficulty doing usual activities and experienced pain were significantly different with the presence of dental caries (*p *= 0.006 and *p *= 0.010, respectively) and bleeding gums (*p *= 0.048 and *p *= 0.014). Finally, those with teeth insecurities also significantly experienced pain than those without teeth insecurities (*p *= 0.031)

**Table 3:** Problems due to conditions of the mouth and teeth by dental caries (self-reported), bleeding gums, and teeth insecurities (self-reported) Note: Teeth insecurities: crooked teeth, gaps between your teeth, stained teeth, chipped/broken teeth, or smell. Median values followed by Inter Quartile Range(IQR) in parentheses. *p-*values for the Mann-Whitney U test.

	Dental caries			Bleeding gums			Teeth insecurities		
	Yes (1-3)	No	p-value	Yes	No	p-value	Yes	No	p-value
Difficulty biting or chewing foods	1 (0, 1)	0 (0, 1)	0.002*	1 (1, 1.5)	0 (0, 1)	0.005*	0 (0, 1)	0 (0, 1)	0.179
Difficulty with speech	1 (0, 2)	0 (0, 1)	0.229	1 (0, 1)	0 (0, 1)	0.027*	0 (0, 2)	0 (0, 1)	0.011*
Dry mouth	1 (0, 2)	0 (0, 1)	0.030*	1 (0, 1.5)	0 (0, 1)	0.080	1 (0, 1)	0 (0, 1)	0.920
Avoided smiling	0 (0, 1)	0 (0,1)	0.182	0 (0, 1.5)	0 (0, 1)	0.070	0 (0, 1)	0 (0, 1)	0.001
Took days off work or school	0 (0, 1)	0 (0, 0)	0.004*	0 (0, 1)	0 (0, 0)	0.045*	0 (0, 0)	0 (0, 0)	0.613
Difficulty doing usual activities	0 (0, 1)	0 (0, 0)	0.006*	0 (0, 1)	0 (0, 0)	0.048*	0 (0, 1)	0 (0, 0)	0.130
Experienced pain	1 (0, 2)	0 (0, 1)	0.010*	1 (0, 2)	0 (0, 1)	0.014*	1 (0, 2)	0 (0, 1)	0.031*

Table 4 presents the variation of attitudes toward oral health and dental care by demographic variables (age and gender) and self-reported oral health issues (dental caries, bleeding gums, and teeth insecurities). Of the various attitude statements, “I value keeping my mouth healthy” was significantly different with both self-reported dental caries and bleeding gums, with students reporting higher median and 25th and 75th percentiles in groups with no dental caries and bleeding gums, respectively (*p *= 0.002; *p *= 0.005). “As I grow old, I accept that I will lose some of my teeth” was significantly different for age group, with 18-24-year-olds reporting lower median values than those who were 25 years or older and the difference being statistically significant (*p *= 0.022). “It is easier to get ahead in life if I have straight bright teeth” was significantly different with bleeding gums while brushing/flossing. Those who experienced bleeding had a higher median value than those who did not (*p *= 0.028).

**Table 4:** Attitudes toward oral health and dental care by age, gender, dental caries, bleeding gums, and teeth insecurities (self-reported) Median values followed by interquartile range (IQR) in parentheses. *p-*values for the Mann-Whitney U test.

	Age			Gender			Dental caries			Bleeding Gums			Teeth Insecurities		
	18-24	≥25		Male	Female		Yes (1-3)	No		Yes	No		Yes	No	
I value keeping my mouth healthy.	5 (4, 5)	5 (4, 5)	0.505	5 (4, 5)	5 (4, 5)	0.916	5 (4, 5)	5 (4.25, 5)	0.002*	4 (4, 5)	5 (5, 5)	0.005*	5 (4, 5)	5 (4, 5)	0.058
Regular visits to the dentist will help keep me healthy.	5 (4, 5)	4 (3.75, 5)	0.353	4 (4, 5)	5 (4, 5)	0.849	5 (4, 5)	5 (4, 5)	0.308	4 (4, 5)	5 (4, 5)	0.301	5 (4, 5)	4 (4, 5)	0.577
As I grow old, I accept that I will lose some of my teeth.	3 (2, 4)	4 (4, 5)	0.022*	4 (3, 4)	3 (2, 4)	0.165	4 (2, 4)	3 (2, 4)	0.374	4 (2, 4)	3.5 (2, 4)	0.513	3 (2, 4)	4 (2, 4)	0.875
I need to see the dentist twice a year.	4 (4, 5)	4 (3, 5)	0.545	4 (4, 5)	4 (3, 5)	0.441	4 (3.5, 5)	4 (4, 5)	0.563	4 (3, 5)	4 (4, 5)	0.461	4 (4, 5)	4 (3, 5)	0.715
It is easier to get ahead in life if I have straight bright teeth.	4 (3, 5)	4 (3, 5)	0.466	3 (2.5, 4)	4 (3, 5)	0.385	4 (3, 5)	4 (3, 5)	0.798	4 (3, 5)	3 (3, 4)	0.028*	4 (3, 5)	4 (3, 5)	0.441

## Discussion

Through this study, we were able to gain a further understanding of oral health behaviors in adolescence and young adulthood and how oral health issues affect OHRQoL and attitudes toward oral health among minority students. Determining students’ oral health history revealed that 63% of those who reported dental caries as adults had experienced caries in adolescence. On average, adolescents experience one missing or decayed tooth, yet Mexican American and Black adolescents are more likely to experience multiple missing teeth and untreated decay [20]. This is significant considering high caries experience as a child is the strongest predictor of dental caries as an adolescent and into adulthood [21]. Furthermore, nearly 70% of Mexican American adolescents and 57% of non-Hispanic Blacks experience dental caries, percentages that are considerably higher among minorities where poverty status also plays a pivotal role before the age of 18 years [22]. In our study, we found that 9% of Hispanics and 32% of African Americans reported untreated dental caries.

Although not significant in our study, 59.3% of students with caries and 56% with bleeding gums had no supervision while brushing their teeth as a child. Likewise, 18-24% of the students were not supervised by their parents while brushing and were allowed to consume sugary foods often. Parental supervision is known to be associated with better oral health, especially in preschoolers, as it can establish adequate toothbrushing habits growing into adolescence and adulthood [23]. In addition to supervision while brushing, dietary choices may influence oral health as an adult since consuming sweets very often as a child was reported by 39% of students with caries in our study. Given that prior studies [23-25] have provided evidence that frequent consumption of sugary sodas is associated with the presence of dental caries, exposure to healthy dietary choices and adequate toothbrushing practices early in life may prompt continued maintenance of these behaviors as an adult and reduce the risk of caries.

Of all the oral health habits, length of brushing was significantly associated with self-reported bleeding gums. Likewise, students who brushed for one minute or less experienced gum bleeding, which may have been due to poor oral hygiene leading to gum disease or the intensity of toothbrushing during a shortened brushing time. Furthermore, longer brushing times of at least two minutes are more effective at removing plaque [26], which is the most common cause of gingival bleeding [27]. Moreover, the intensity and duration of toothbrushing is a practice learned in childhood that ultimately becomes more difficult to modify as an individual ages, especially if they do not recognize a need to change the practice.

In addition, the current study’s findings indicate several items within OHRQoL to be associated with self-reported oral health issues. Dental caries, for example, were significantly associated with difficulty biting/chewing, pain, dry mouth, interference in daily activities, and days missed at school or work. Similarly, a study of Brazilian adolescents found dental caries to be significantly associated with difficulty eating and interference in daily activities [28]. Avoiding smiling and trouble sleeping (*p* = 0.018) were also found to have significant associations with dental caries in the study [28]. The current study found bleeding gums to be associated with difficulty biting/chewing, pain, speech, interference in daily activities, and days missed at school or work. Few studies have evaluated the specific impact of gingival bleeding on these factors; however, Thirunavukkarasu et al. found poor self-rated oral health to be significantly associated with pain in the gums or mouth (*p* = 0.012) [29]. Moreover, poor oral health in general has been associated with painful aching [26-27] and frequent discomfort while eating [28-30]. Teeth insecurities such as crooked, stained teeth or gaps between teeth were significantly associated with pain, avoidance of smiling, and speech difficulty, and similar findings were seen in other studies [29-30]. Furthermore, many individuals with poor oral health reported feeling embarrassed occasionally to very often [29-30], while some felt self-conscious and anxious due to their oral health [29].

Attitudes toward oral health and dental care were also examined by demographics and self-reported oral issues, and the findings suggest that the presence of dental caries and bleeding gums was significantly associated with lower attitude scores in valuing to keep mouth healthy, which corroborates with previous evidence [8]. The attitude regarding “It is easier to get ahead in life if I have straight bright teeth” was lower among those with bleeding gums and is consistent with the findings of Ericsson et al., which indicated that those with poor oral health have negative attitudes and perceptions about oral health [8]. Furthermore, respondents in the age group 18-24 years scored lower than age group 25 years or older in attitude pertaining to loss of teeth with age. Percentage distributions of respondents agreeing to attitude toward oral health and dental care were lower for all the statements than the percentages reported in an oral health and well-being survey in the United States [31].

Nearly 40-43% of the students in the current study reported one to three untreated dental caries and gums that bled. One potential attribute to the findings may be that oral health in minority populations has historically shown a greater rate of dental caries and fewer dental visits than in other communities [32]. Although oral health has improved over time, many in the United States still lack adequate access to quality dental care [24]. Florida has left many individuals without access to preventive care and treatment for oral health issues due to less than three counties having enough dentists to serve all its residents [14]. Given this, ensuring more minority college students can practice effective oral hygiene is not only dependent on establishing oral health knowledge and practices as an adolescent but also increasing opportunities for minority populations to receive quality preventative care.

This study relied on self-reported oral health issues to examine oral health inequities experienced by minority college students. Furthermore, focusing this study on the university’s minority population will contribute to the few research studies that have been done on minority college students and oral health. Likewise, oral health should be acknowledged with equal significance to general health in terms of health education. Although it is believed that minority students are knowledgeable about oral health, there are many factors beyond those studied that could be barriers to healthy oral behaviors in minority students.

Due to a lack of studies examining oral health attitudes and behaviors among minority college students, the current study’s findings may be considered novel but not without limitations. First, recall bias is plausible since the study utilized a survey that relied upon self-reported behaviors and dental conditions. In addition, the sample may not be truly representative of the minority student population. Finally, the results may be limited given the questionnaire focused on dental caries and bleeding gums as the primary conditions signifying poor oral health. In order to achieve a greater understanding of how oral health impacts behaviors and quality of life in minority students, future studies should include objective and expansive criteria on what constitutes poor oral health.

## Conclusions

The study highlights experiencing pain, avoiding smiling, and difficulty with chewing and speech being affected by dental caries, bleeding gums, and teeth insecurities. Minority students with oral health problems also reported lower attitude scores regarding valuing oral health, retaining teeth with age, and regular dental visits. A substantial portion of the students brushed for one minute or less and experienced dental caries as adolescents. Improving oral health education resources for minorities and free preventative care beginning in adolescence may help improve oral health practices among minorities over their lifetime. Furthering education on the importance of preventative oral care and providing financial equality of dental care would ideally improve oral health among minority populations.
